# Rapid detection of *Mycobacterium tuberculosis* DNA and genetic markers for Isoniazid resistance in Ziehl-Neelsen stained slides

**DOI:** 10.1590/0074-02760190407

**Published:** 2020-04-17

**Authors:** Graziele Lima Bello, Franciele Costa Leite Morais, Sheile Pinheiro de Jesus, Jonas Michel Wolf, Mirela Gehlen, Isabela Neves de Almeida, Lida Jouca de Assis Figueiredo, Tainá dos Santos Soares, Regina Bones Barcellos, Elis Regina Dalla Costa, Silvana Spíndola de Miranda, Maria Lucia Rosa Rossetti

**Affiliations:** 1 Universidade Luterana do Brasil, Programa de Pós-Graduação em Biologia Celular e Molecular Aplicada à Saúde, Canoas, RS, Brasil; 2 Universidade Federal do Rio Grande do Sul, Programa de Pós-Graduação em Pneumologia, Porto Alegre, RS, Brasil; 3 Universidade Federal de Minas Gerais, Faculdade de Medicina, Laboratório de Pesquisa em Micobactérias, Belo Horizonte, MG, Brasil; 4 Universidade Luterana do Brasil, Graduação em Biomedicina, Canoas, RS, Brasil; 5 Secretaria do Estado do Rio Grande do Sul, Centro de Desenvolvimento Científico e Tecnológico, Porto Alegre, RS, Brasil; 6 Universidade Federal do Rio de Janeiro, Programa de Pós-Graduação em Clínica Médica, Rio de Janeiro, RJ, Brasil; 7 AstraZeneca do Brasil, Cotia, SP, Brasil

**Keywords:** Mycobacterium tuberculosis, Isoniazid resistance, genotyping, real-time PCR

## Abstract

**BACKGROUND:**

Early diagnosis of tuberculosis (TB) and identification of strains of *Mycobacterium tuberculosis* resistant to anti-TB drugs are considered the main factors for disease control.

**OBJECTIVES:**

To standardise a real-time polymerase chain reaction (qPCR) assay technique and apply it to identify mutations involved in *M. tuberculosis* resistance to Isoniazid (INH) directly in Ziehl-Neelsen (ZN) stained slides.

**METHODS:**

Were analysed 55 independent DNA samples extracted from clinical isolates of *M. tuberculosis* by sequencing. For application in TB diagnosis resistance, 59 ZN-stained slides were used. The sensitivity, specificity and Kappa index, with a 95% confidence interval (CI95%), were determined.

**FINDINGS:**

The agreement between the tests was, for the *katG* target, the Kappa index of 0.89 (CI95%: 0.7-1.0). The sensitivity and specificity were 97.6% (CI95%: 87.7-99.9) and 91.7% (CI95%: 61.5-99.5), respectively. For *inhA*, the Kappa index was 0.92 (CI95%: 0.8-1.0), the sensitivity and specificity were 94.4% (CI95%: 72.7-99.8) and 97.3% (CI95%: 85.8-99.9), respectively. The use of ZN-stained slides for drug-resistant TB detection showed significant results when compared to other standard tests for drug resistance.

**MAIN CONCLUSIONS:**

qPCR genotyping proved to be an efficient method to detect genes that confer *M. tuberculosis* resistance to INH. Thus, qPCR genotyping may be an alternative instead of sequencing.

Drug-resistant and multidrug-resistant tuberculosis (DR/MDR-TB) has increased worldwide, demanding the development of new drug resistance detection assays. The main drugs used in the first-line treatment of TB are Isoniazid (INH) and Rifampicin (RIF) and resistance to both drugs means MDR-TB. Until recently, RIF was considered a marker of MDR-TB, because frequently that resistance is associated with INH resistance. There have been recurrent studies showing the agent *Mycobacterium tuberculosis* resistant to INH and sensitive to RIF; meaning a risk factor for unfavorable outcomes.^1^

Globally, monoresistance to INH presented in 2014 an overall mean of 9.5% in new cases and 14% in previously treated TB. It is estimated that more than 1 million people develop INH resistant, each year.^2^ Nevertheless, routine tests for INH resistance are not used as frontline tests in most scenarios.^3^ In Brazil, the most used test for the rapid screening of TB drug resistance (at main health centres) is the Gene Xpert MTB/RIF, however, it only detects resistance to RIF. Commercial molecular hybridisation tests to detection INH resistance, such as GenoType MTBDR*plus* ^R^ line probe assays (Hain Lifescience, Nehren, Germany), are sensitive and specific, but, in Brazil, only a few sites use it routinely and the clinical impact of this test has not been evaluated.^4^

Resistance to INH is associated with a wide variety of mutations that affects one or more genes, such as *katG*, *inhA*, *ahpC* genes. In general, mutations in *katG* and *inhA* genes are found in 75-85% of the isolates resistant to this drug.^5^ The most common mutation in the *katG* gene arises at codon 315 by replacing the serine amino acid (AGC) to threonine (ACC), with a decrease in the action of catalase, important for activating the drug.^6^ In several studies around the world, the frequency of INH resistant strains varies from 50 to 100% with the mutation at codon 315 of the *katG* gene.^7^ The *inhA* gene encodes the enoyl-ACP reductase (NADH) fatty acid carrier protein, which is essential in the synthesis of mycolic acids in the cell wall of the bacterium. The mutation of the *inhA* gene modifies the enzyme, which loses affinity for NADH, resulting in resistance to INH.^8^

One of the limitations of performing molecular tests is the safe storage and transportation of sputum samples (potentially infectious) from remote health centres to central laboratories. In addition, the high cost and proper transportation logistics for this type of sample, as well as the return of test results to the source laboratory and to the patient can also be a challenge. In view of this, ZN-stained slides (which is the first test commonly performed in most peripheral-level laboratories and health centres in low-income countries), would be readily available and could be the ideal tool for safe system transportation of sputum, analysis of molecular tests and therefore timely management of TB patients.^9^

Thus, in the face of the need for further studies on the identification of resistance to INH and the development more cost-effective technologies, this study aimed to standardise and identify mutations involved with *M. tuberculosis* INH resistance by real-time polymerase chain reaction (qPCR) genotyping assays, using DNA extracted from clinical isolates (standardisation) e smear slides samples (application in TB diagnosis resistance).

## MATERIALS AND METHODS

*Standardisation of TaqMan* ^*R*^ *qPCR genotyping assays to the detection of INH resistance in culture of M. tuberculosis* - This study was performed using a total of 55 independent DNA samples extracted from clinical isolates of *M. tuberculosis*, according to described by Van Soolingen et al.,^10^ Samples were stored at -80ºC in the Molecular Biology Laboratory, at the Centre for Scientific and Technological Development (CDCT) of the State Health Secretariat (SES), located in the city of Porto Alegre, Southern Brazil. These DNA samples were already characterised by the sequencing of genes regions involved with INH resistance).^7^ DNA was quantified by spectrophotometry using the SpectraMaxPlus^R^ (Eppendorf) spectrophotometer according to the manufacturer’s instructions. The concentrations of 100, 50, 20 and 10 ng/µL were evaluated. The amplifications that showed fluorescence signals with sigmoidal curves plus detection characteristics by qPCR were the samples quantified with a final DNA concentration of 20 ng/µL. This final concentration was ideal for qPCR identification.

Previously, all the 55 isolates used in the present study were confirmed as containing acid-fast bacilli by microscopy detection on ZN-stained slides.^11^ Standard bacteriological and biochemical tests were performed for differentiation of species within the *M. tuberculosis* complex (MTBC) and Mycobacteria other than tuberculosis (MOTT), including biochemical testing for niacin, paranitrobenoic acid (PNB) and tiofeno-2-carboxylic acid hydrazine (TCH).^11^ Subsequently, the isolates were submitted to drug susceptibility testing (DST) using the Bactec^TM^MGIT^TM^960 system, according to the manufacturer.^12^

*Application of TaqMan* ^*R*^ *qPCR genotyping assays to the detection of INH resistance in DNA extracted from Ziehl-Neelsen stained slides* - For the application of qPCR assays in clinical samples, 59 smear slides were used from the Mycobacterial Laboratory, School of Medicine, Federal University of Minas Gerais (UFMG/Brazil). These specimens had the following tests: sputum smear microscopy (ZN), solid mycobacterial culture from Lowestein Jensen, phenotypic species identification tests, anti-TB drug tests,^11^ GeneXpert MTB/RIF,^13^ and GenoType MTBDR*plus* ^R^ - Hain Tape.^14^ DNA extraction from slides was performed using 5% Chelex from a protocol adapted from Van Der Zanden,^15^ as follows: 100 μL of ultrapure water was added over the smear slide to facilitate detachment of the sample; then a clean slide was used to scrape the material. This was transferred to another microtube, where the 5% Chelex reagent solution was subsequently added. The samples were then incubated in a thermoblock at 56°C for 30 min and then at 100ºC for 10 min for cell lysis. The microtubes were centrifuged at 14,000 rpm for 15 min, and the supernatant was transferred to another microtube. To improve the quality, each DNA sample was subjected to a purification step using silica columns and washed with specific buffers (adapted from Boom et al.).^16^

*Real-time PCR* - The qPCR analysis was performed using in a *StepOne Real-time PCR System* (AB Applied Biosystems) and the amplified products were detected by the TaqMan^R^ detection system (AB Applied Biosystems). The reaction was standardised in a total volume of 20 μL, containing 10 μL of the kapa master mix, 1 μL of *primers* solution and probes (AB Applied Biosystems) at a concentration of 20X. 2 μL (10 ng/uL) of DNA was added in the mix. The control used as wild type (WT) profile (for both genes) was the DNA of the *M. tuberculosis* H37Rv reference strain. As a negative reaction control, the PCR mix containing nucleus-free ultrapure water was used. Amplification conditions were activation of the enzyme at 95ºC for 10 min (heating stage), 40 cycles of denaturation at 95ºC for 15 s and annealing and extension at 60ºC for 1 min. There were pre-PCR and post-PCR steps of 60ºC for 30 s. These assays for detection of gene target polymorphisms have been customised by Applied Biosystems/Thermo Fisher Scientific (4332077-Custom TaqMan SNP genotyping assays non-human, SM). Large sequences of “base” fragments corresponding to the target regions used for identification of INH resistant *M. tuberculosis* (*katG* 315 G/C and *inhA* -15 C/T) were sent for the synthesis of the assays. The analysis of the results was performed with a tool offered by the manufacturer: Thermo Fisher Cloud (https://www.thermofisher.com/search/results?query=cloud&focusarea=Search%20All), which provides more options for the evaluation of the results.

*Statistical analyses* - Data were analysed using the Statistical Package for Social Sciences (SPSS, version 23.0, Chicago, IL). To evaluate the agreement between the results of the tests performed, the Kappa index was calculated (in pairs). The sensitivity, specificity, Kappa index and receiver operating characteristic (ROC) curve for the analysis of the accuracy level of the genotyping tests, with a 95% confidence interval, were also evaluated.

*Ethical aspects* - This study was approved by the research ethics committee of the School of Public Health/Health Department ESP/SES/RS (approval number: 1.607.182) and Federal University of Minas Gerais (number: CAAE: 02232412.7.1001.5149).

## RESULTS

*Standardisation procedures* - The genotypic profile obtained by sequencing characterised the samples in 9.11% [(5/55); WT (katG)/MUT (inhA)], 12.72% [(7/55); WT (katG)/WT (inhA)], 52.72% [(29/55); MUT (katG)/WT (inhA)] and 25.45% [(14/55); MUT (katG)/MUT (inhA)].

*Genotyping of katG gene (315 G/C)* - Of the 55 DNAs extracted from INH resistant cultures, 43 mutated (MUT) and 12 WT were detected in the region of the gene, according to the characterisation by sequencing. Genotyping by qPCR detected 42 MUT (42/43) and 11 WT (11/12) samples. The agreement value of the Kappa index was 0.89 (CI95%: 0.7-1.0) (agreement considered excellent according to the classification quality). The sensitivity and specificity were 97.6% (CI95%: 87.7-99.9) and 91.7% (CI95%: 61.5-99.5), respectively (Table I). In the detection graphs, the analysis of the curves allows us to evaluate the genotypic profile of each template, as well as to discriminate the distribution of the clusters [see Supplementary data (Fig. 1)]. The ROC curve was used to evaluate the sensitivity (or rate of true positives) *versus* specificity (false positive rate), in the other words, it is the representation of a visual index of the accuracy of the assay [see Supplementary data (Fig. 2)]. The area under the curve, which is a statistical summary for the determination of the level of test accuracy, was 0.94 (reference = 1.0).

**TABLE I:** Performance of *katG* and *inhA* genes genotyping by real-time polymerase chain reaction (qPCR) in clinical isolates

Assay	Sequencing (MUT)	Sequencing (WT)	Total	Sensitivity (CI 95%)	Specificity (CI 95%)	Kappa index (CI 95%)
qPCR *katG* (MUT)	42	1	43	97.6	91.7	0.89
qPCR *katG* (WT)	1	11	12	(87.7 - 99.9)	(61.5 - 99.7)	(0.7 - 1.0)
Total	43	12	55			
qPCR *inhA* (MUT)	17	1	18	94.4	97.3	0.92
qPCR *inhA* (WT)	1	36	37	(72.7 - 99.8)	(85.8 - 99.9)	(0.8 - 1.0)
Total	18	37	55			

CI: confidence interval; MUT: mutated; WT: wild type.

*Genotyping of inhA gene (-15 C/T)* - As from 55 extracted DNAs, the sequencing identified 18 MUT and 37 WT samples. Genotyping by qPCR assay identified 17 MUT and 36 WT samples (one discordant sample for each profile). The value of the Kappa index was 0.92 (CI95%: 0.8-1.0) (agreement considered excellent). Sensitivity and specificity were 94.4% (CI95%: 72.7-99.8) and 97.3% (CI95%: 85.8-99.9), respectively (Table I). Fig. 3, in Supplementary data, shows the plots from the genotyping experiments. The ROC curve [see Supplementary data (Fig. 4)] showed test accuracy (area under curve) of 0.96 (reference = 1.0). As genotyping for the previous target, the *inhA* target exhibited consistent performance.

*FN-stained slides* - All negative smear slides were culture-matched, as well as with GeneXpert MTB/RIF (TRM - Rapid Molecular Test). The qPCR detected (from the negative smear slides and culture) a WT profile sample for both genes studied. The agreement of qPCR with negative culture was 96.7%. On smear-positive slides that also had a positive culture test, the DST and the GenoType MTBDR*plus* ^R^ were performed. The first test is based on phenotypic identification and the second, on gene analysis, therefore, in DST, only antimicrobial resistance (INH) can be analysed. A comparison of gene analysis is only possible when comparing qPCR *versus* GenoType MTBDR*plus* ^R^. The Kappa index for the agreement between the last two tests cited (valid cases) in relation to the *katG* and *inhA* genes was 0.82 and 1, respectively. If we compare the samples for INH drug sensitivity and resistance (DST) *versus* GenoType MTBDR*plus* ^R^/qPCR), the obtained Kappa indices were 0.69 and 0.25, respectively [see Supplementary data (s 5, 6, 7)].

A comparison was also performed between the positive FN-stained slides quantified as + (1 to 10 bacilli by field in 50 observed fields), ++ (10 to 99 bacilli in 100 observed fields), +++ (on average more than 10 bacilli by field in 20 observed fields), 1 to 9 bacilli in 100 observed fields)^11^ and the threshold cycling (Cts) discriminated by qPCR (Table II). In some cases, the smear slides that had the most bacilli in observed fields, had lower Cts, in others [slide 10, 11 and 18 (Table II)], there was not the same agreement. The microscopy of the ZN-stained slides [slide 1 (Table II)] did not reveal acid-fast bacilli, although the sample contained in the slide provided sufficient DNA for amplification by qPCR identification.

**TABLE II:** Comparison between the Ziehl-Neelsen (ZN)-stained slides classification and the threshold cycling (Cts) detected samples by real-time polymerase chain reaction (qPCR) assays

Samples	ZN slides	qPCR (Cts katG)	qPCR (Cts inhA)
1	NEG	WT (31.01)	WT (30.84)
2	++	WT (32.35)	WT (35.90)
3	+	WT (33.20)	WT (33.64)
4	3 BACILLI / 100 FIELDS.	UNDETERMINED	UNDETERMINED
5	3 BACILLI / 100 FIELDS.	UNDETERMINED	UNDETERMINED
6	+++	WT (32.35)	WT (35.53)
7	3 BACILLI / 100 FIELDS.	WT (35.23)	WT (35.15)
8	+++	WT (28.06)	WT (28.41)
9	3 BACILLI / 100 FIELDS.	UNDETERMINED	UNDETERMINED
10	+	WT (33.19)	WT (32.55)
11	++	WT (36.27)	WT (36.99)
12	++	UNDETERMINED	UNDETERMINED
13	+	WT (37.89)	WT (36.95)
14	+	WT (32.28)	WT (32.01)
15	+	WT (32.89)	WT (35.54)
16	+	MUT (29.81)	WT (30.53)
17	+++	WT (36.57)	WT (36.20)
18	+++	WT (33.96)	WT (36.16)
19	4 BACILLI / 100 FIELDS.	WT (32.72)	WT (35.91)
20	+	WT (33.46)	WT (34.61)
21	+	MUT (33.95)	WT (36.00)
22	+++	WT (32.81)	WT (34.29)
23	+++	WT (32.15)	WT (33.01)
		WT (33.79 ± 2.33)	WT (35.71 ± 0.84)
		MUT (31.88 ± 2.92)	-

NEG: negative; MUT: mutated; WT: wild type; +: 10 to 99 bacilli in 100 observed fields; ++: 1 to 10 bacilli by field in 50 observed fields; +++: on average more than 10 bacilli by field in 20 observed fields. 1 to 9 bacilli in 100 observed fields. Median ± standard deviation.

## DISCUSSION

Monoresistance to INH has become increasingly common over the years. Studies have found high rates of resistance for this drug, and there is still no consensus on how patients can be treated. Furthermore, the treatment outcome is most likely unfavorable and failure to identify an INH-resistant isolate is equivalent to losing the efficacy of one of the drugs and compromising the basic regimen of treatment.^17^ The lack of adequate treatment to combat an INH resistant isolate could also be inducing gene-level changes (mutations), generating MDR clones, since resistance to RIF comes in sequence.^2^ The qPCR assays performed in this study identified the most known mutations in genetic regions associated with INH resistance (*katG* 315 G/C and *inhA* -15 C/T; GenBank: (MG995339, MG995338, MG995265 e CP023597 - National Centre for Biotechnology Information - NCBI - https: //www.ncbi. nlm.nih.gov/genbank/). It is important to consider that the mutations associated with INH are more complex and occur in many *M. tuberculosis* genes. However, the most used genes as markers, according to studies, such as a recent systematic review (Seifert et al.),^18^ which evaluated 11.411 *M. tuberculosis* isolates in 49 countries (January 2000 to August 2013), are the *katG* (315 G/C) and the *inhA* (-15 C/T) promoter region. Confirming these findings, we also have the study of Monteserin et al.,^19^ who evaluated the same markers to analyse the drug resistance profile of INH and the distribution of clusters in Argentina, as well as the study of Lopez-Avalos et al.,^20^ which used the same markers to identify the resistance already described.

Regarding the agreement of the standardisation results, the sensitivity, specificity, Kappa index and ROC curve are evaluated in most of the studies that use the analysis of diagnostic tests and/or comparison of results between techniques (GeneXpert MTB/RIF, GeneXpert MTB/RIF Ultra, Genotype MTBDRplus, TB-SPRINT, and others).^21^ Concerning the discrepancies in the agreement of the tests, we believe that it is due to the alternative molecular mechanisms of resistance, many of which are still unknown, or even that they present a rare genotypic profile, found in a few strains of the *M. tuberculosis* complex.^22^ Another important bias to consider is that some tests have different detection methods, such as DST and GeneXpert MTB/RIF (microbiological *versus* molecular detection), therefore, some comparisons allowed statistical calculations with greater equity, but others had more restricted analyses to the detection method.^21^ ^,^ ^23^

With regard to the diagnosis of drug-resistant TB in clinical sputum samples, it is known that in low-income countries there are frequent obstacles in transporting and storing samples from peripheral regions to reference laboratories performing molecular analyses, restricting surveillance and the population of the real scenario of the disease.^24^ Facing this situation and based on the literature, smear microscopy slides (first test most commonly used for screen for pulmonary TB; culture and DST methods are not routinely used as they require more modern equipment and larger biosafety structure, that are only available from reference laboratories) can be a safe way to transport sputum samples from peripheral sites to central laboratories that perform molecular tests for TB, increasing the speed of diagnosis and promoting better patient management.^9^

Thus, we showed that DNA extracts from ZN-stained slides may be used as a template for the amplification of *M. tuberculosis* DNA by qPCR technique. Similar results were also obtained by Gonçalves et al.,^25^ who used the same DNA extraction technique for detection by qPCR assays. De Almeida et al.,^26^ evaluated six different techniques for *M. tuberculosis* DNA extraction, including DNA extraction by Chelex from cultures of microorganism and FN-stained slides. One of the results obtained was that the method using the Chelex + NP-40 for DNA extraction reagents provided an adequate amount of interference-free DNA. Thus, according to the Rakotosamimanana et al.^9^ results, our study allowed the use of smear slides as a source of DNA extraction for the diagnosis of TB and to perform some additional analyses for drug resistance evaluation.

Smear microscopy slides are known to be simple, fast and less costly for the initial diagnosis of TB, however, sensitivity and specificity still remain low.^27^ These measures are also dependent on the quality of the smear performed as well as the training of the microscopy analyser. We found the presence of *M. tuberculosis* DNA in patient sample 1 (Table II), however, the presence of the bacillus was not visualised on microscopy.^28^ Combining the FN-stained slide with bacillus DNA detection and drug resistance testing increases the effectiveness of the analyses and makes them more cost-effective.^27^

Evaluating the results, genotyping analyses in *M. tuberculosis* strains could contribute significantly to disease control, indicate possible epidemiological associations, detect suspicious outbreak information and cross-contamination in laboratories, and even distinguish between reinfections exogenous and endogenous reactivation during retreatment.^29^ Although the use of sequencing is considered the gold standard for resistance detection, recent studies have shown that the use of qPCR genotyping is widely used in molecular diagnostics as well as in resistance profile identification studies due to its specificity, the high degree of automation and good reproducibility.^30^

In the present study, part of the experiments was carried out with DNA extracted from clinical isolates collected from Southern Brazil (standardisation step) and, the other part, performed with DNA extracted from ZN-stained slides collected from Southeast Brazil, used for the application in TB diagnosis resistance. This is the main limitation of this study; however, our findings are important for a better understanding of the diagnosis of patients infected with strains of *M. tuberculosis* resistance to INH. Future studies may consolidate our data, thus allowing better therapeutic management for these patients.

*In conclusion* - Compared to sequencing, our method showed similar sensitivity and specificity to detect genes that confer *M. tuberculosis* sensitivity or resistance to INH. The results suggest that molecular tests can be used for the rapid detection of drug-resistant TB, as FN-stained slides can be considered an effective tool for facilitating DNA extraction from sputum samples, facilitating, bypassing difficulties such as safe storage and transport of samples, also facilitating the diagnosis of TB in peripheral areas. However, further studies with a larger number of samples are still needed to validate the technique and include in the future another TaqMan^R^ assay for the assessment of resistance to RIF.
