# Supplementary material for: Rapid detection of Mycobacterium tuberculosis DNA and genetic markers for Isoniazid resistance in Ziehl-Neelsen stained slides
Source: Mem Inst Oswaldo Cruz. 2020 Apr 17;115:e190407. doi: 10.1590/0074-02760190407 (PMC7164399; doi:10.1590/0074-02760190407)
Supplement: Supplementary file 1 [file 1678-8060-mioc-115-e190407-s.pdf]

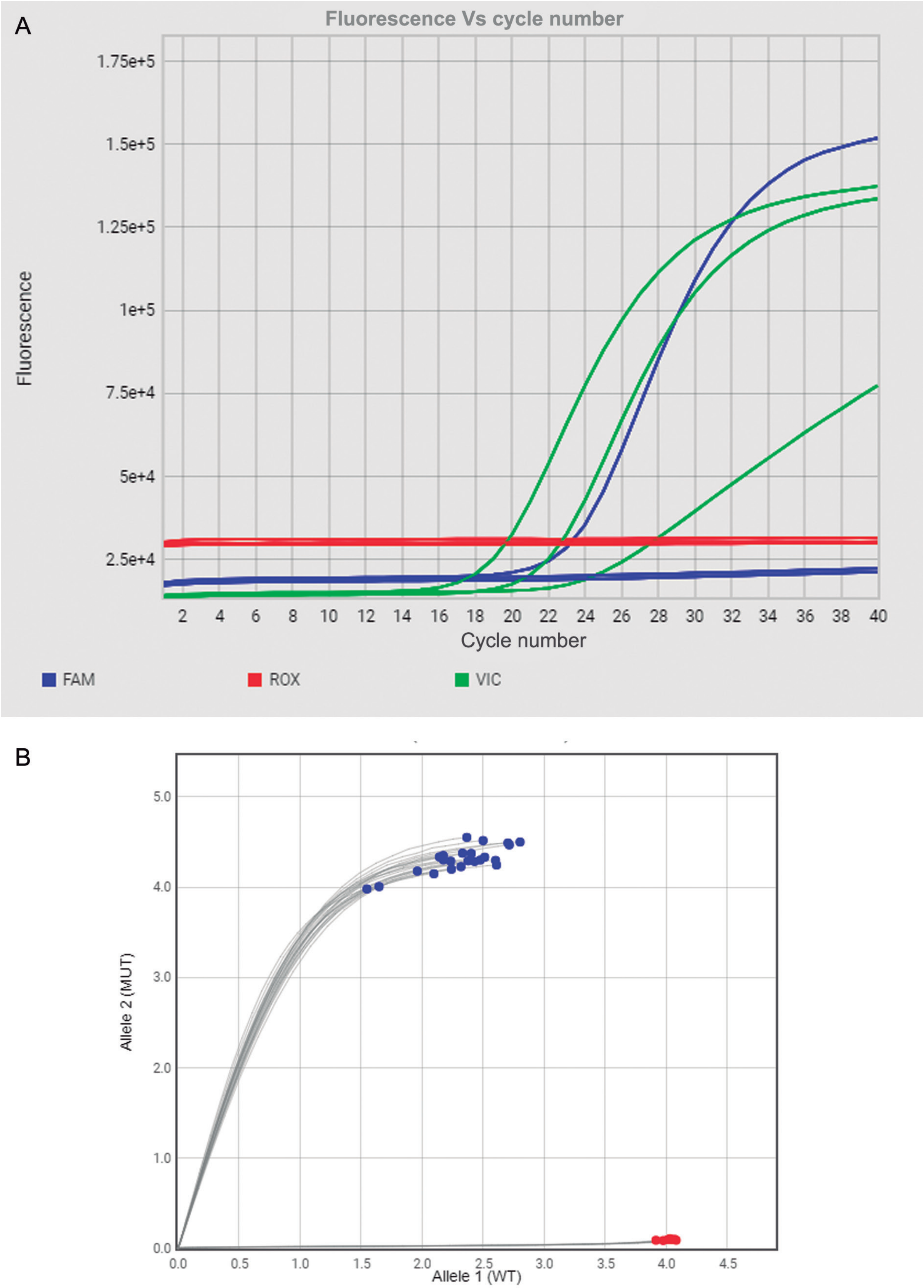

Fig. 1: genotyping profiles for the *katG* target. (A) Multicomponent plot of clinical isolates samples. In this assay, the FAM (blue) fluorophore represents the mutated (MUT) genotype and the VIC (green) fluorophore represents the wild type (WT) genotype. (B) Plot of clusters discrimination.

cc BY

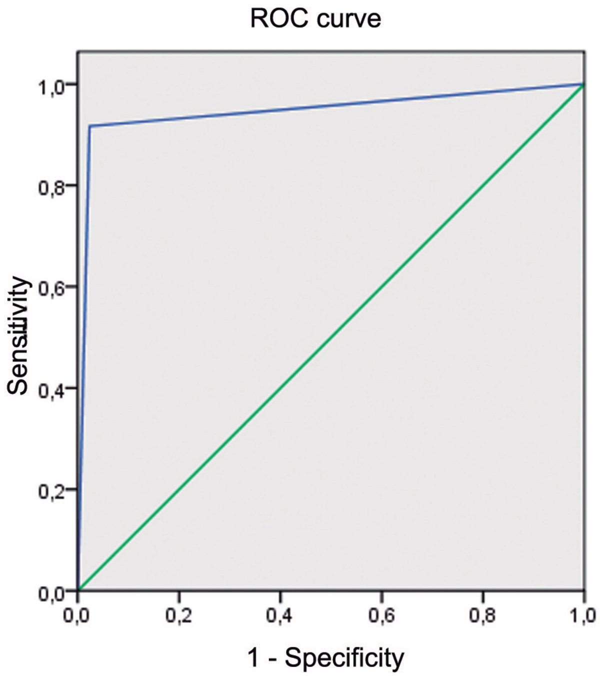

Fig. 2: receiver operating characteristic (ROC) curve (sensitivity *versus* specificity) to discriminate the accuracy level of *katG* target genotyping compared to sequencing.

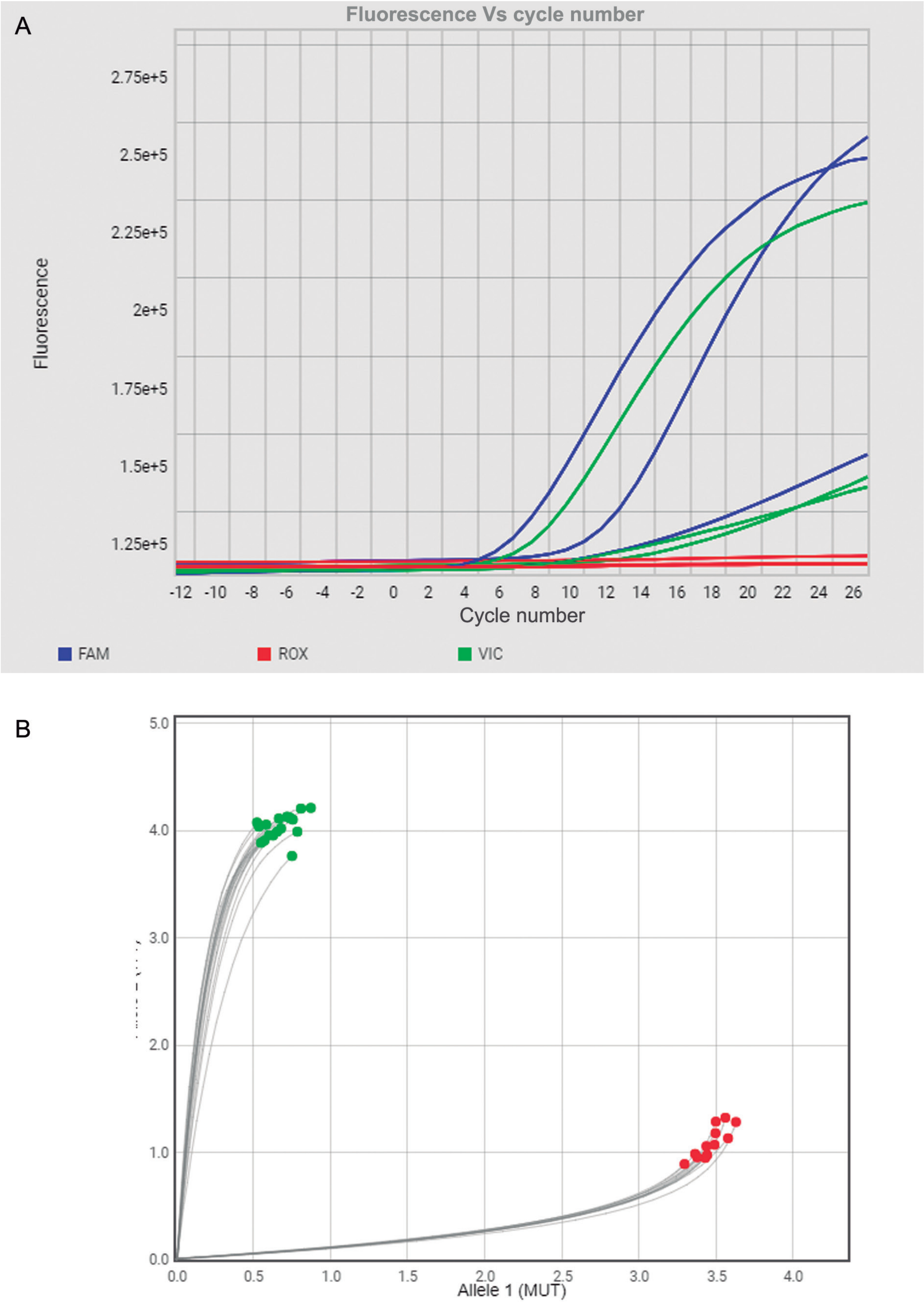

Fig. 3: genotyping profiles for *inhA* target. (A) Multicomponent plot of clinical isolates samples. In this assay, the FAM (blue) fluorophore represents the wild type (WT) genotype and the VIC (green) fluorophore represents the mutated (MUT) genotype. (B) The plot of clusters discrimination.

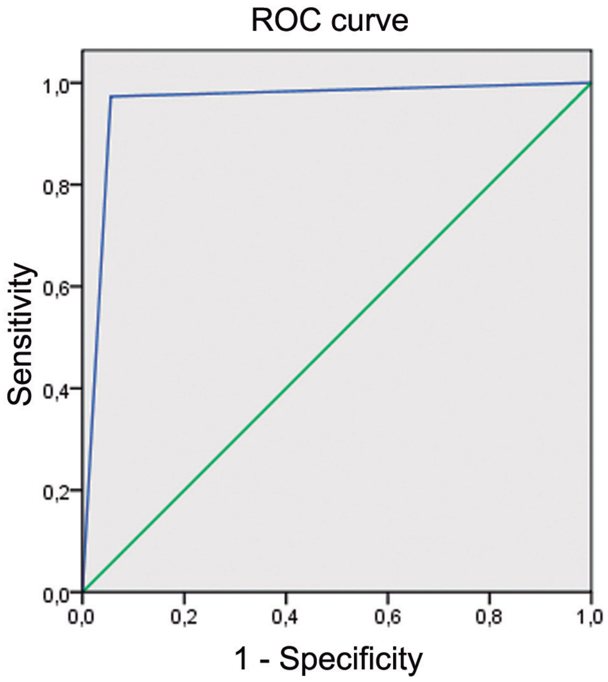

Fig. 4: receiver operating characteristic (ROC) curve (sensitivity *versus* specificity) to discriminate the accuracy level of *inhA* target genotyping compared to sequencing.

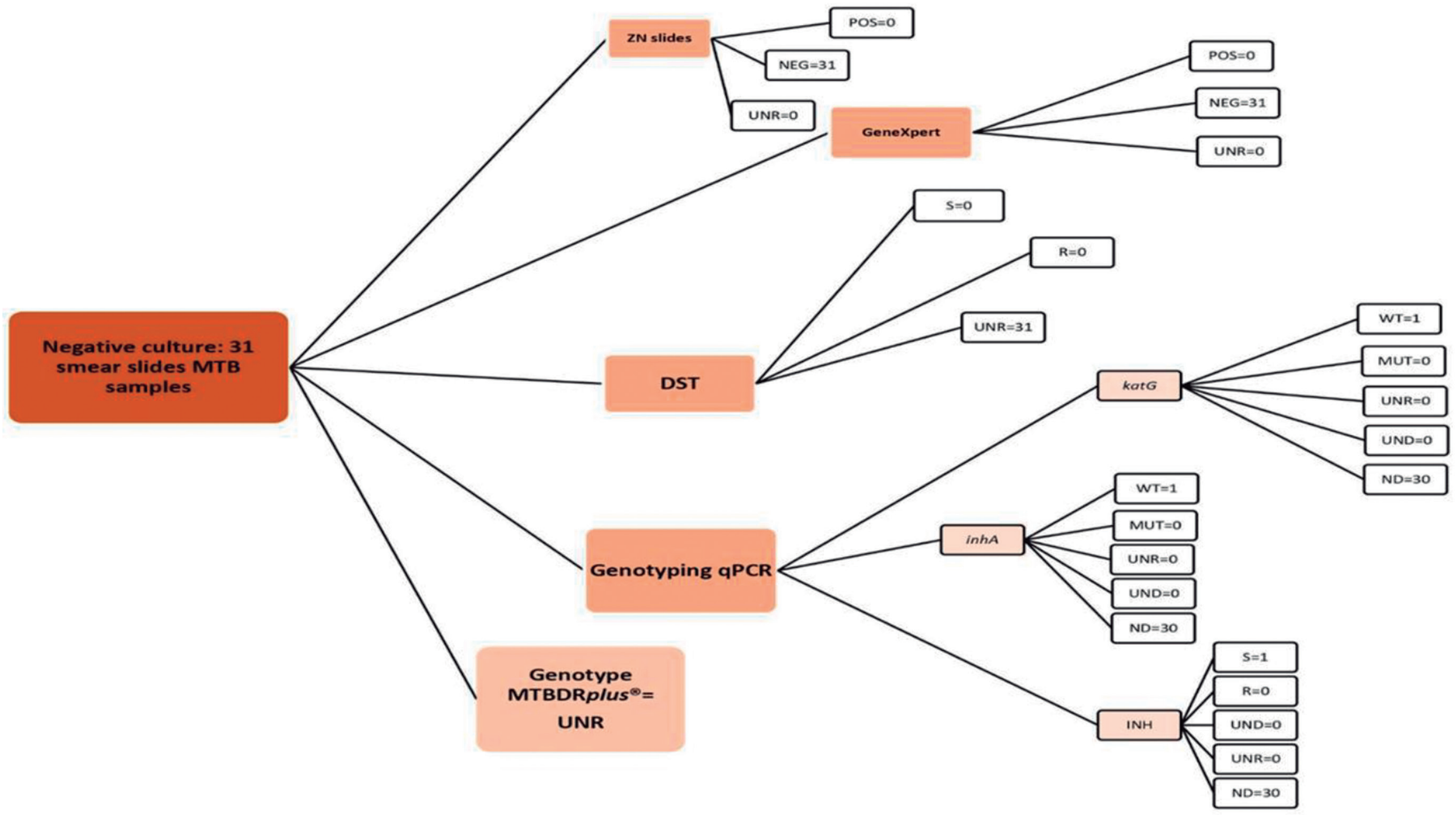

Fig. 5: flowchart of analyses performed on smear slides with negative *Mycobacterium tuberculosis* culture. ZN slides: Ziehl-Neelsen stained slides; WT: wild type; MUT: mutated; POS: positive; NEG: negative; ND: not detected; UND: undetermined; UNR: unrealised; S: sensitive; R: resistant; DST: drug susceptibility testing; qPCR: quantitative polymerase chain reaction; INH: Isoniazid.

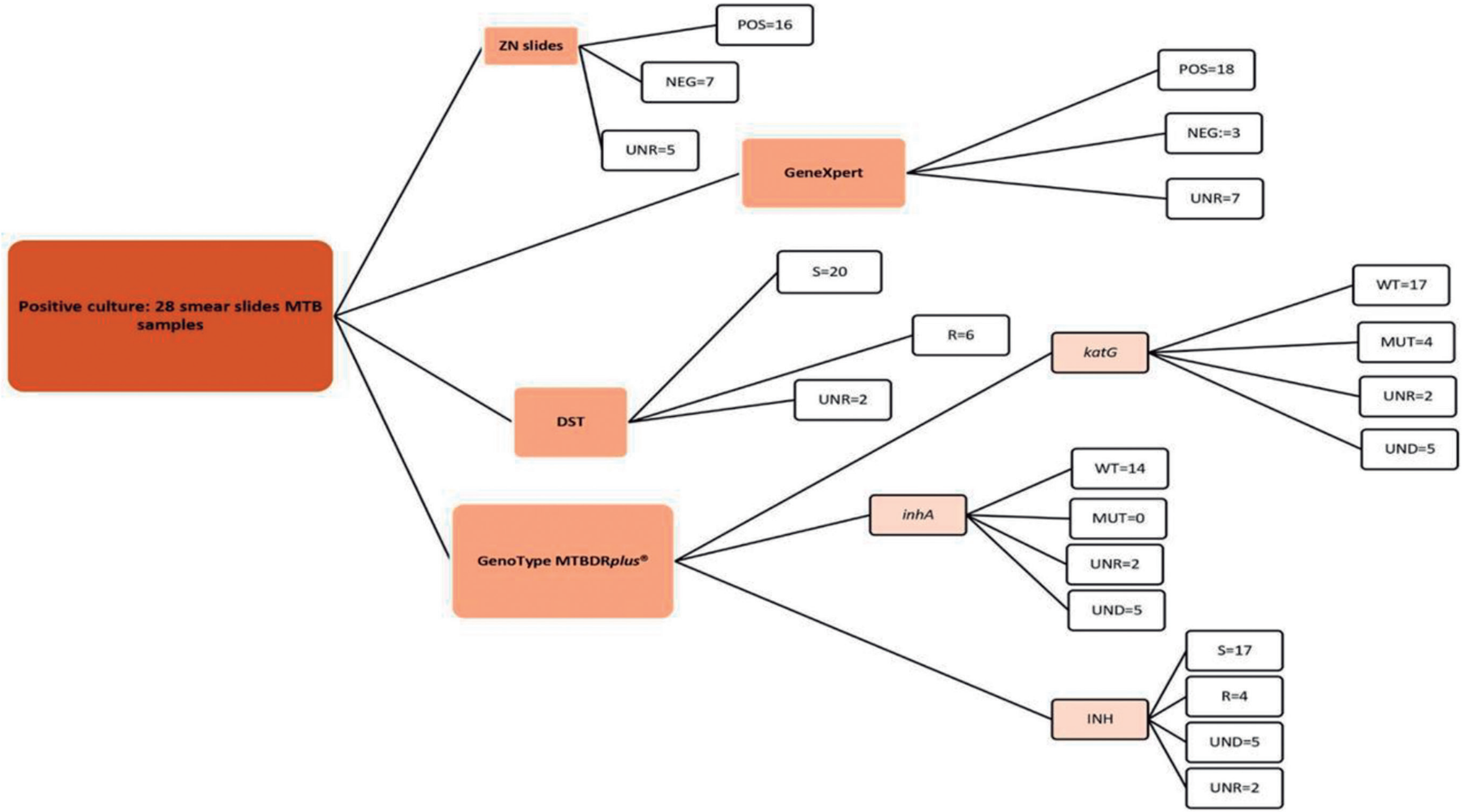

Fig. 6: flowchart of analyses performed on smear slides with positive *Mycobacterium tuberculosis* culture. ZN slides: Ziehl-Neelsen stained slides; WT: wild type; MUT: mutated; POS: positive; NEG: negative; ND: not detected; UND: undetermined; UNR: unrealised; S: sensitive; R: resistant; DST: drug susceptibility testing; qPCR: quantitative polymerase chain reaction; INH: Isoniazid.

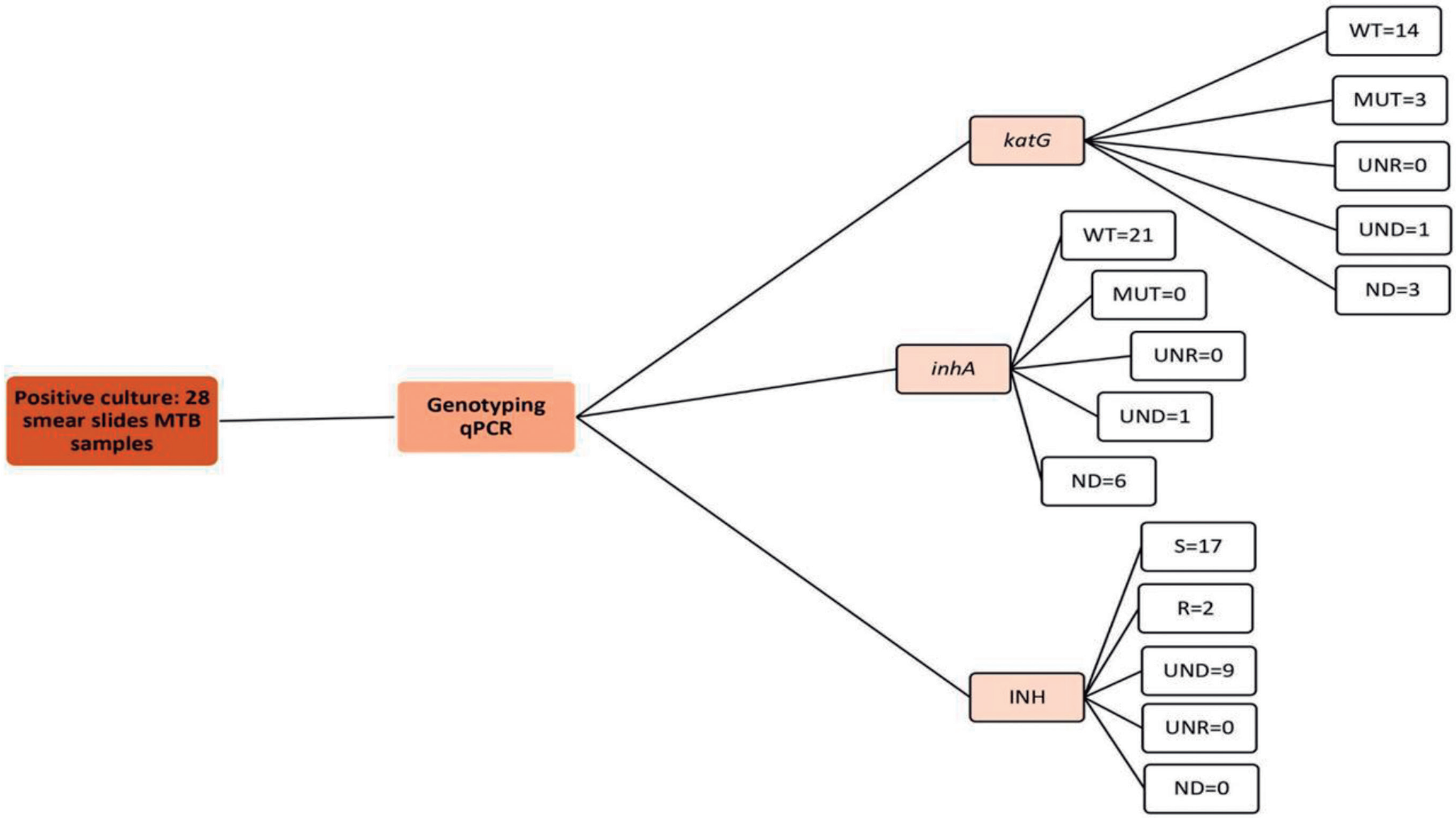

Fig. 7: flowchart of analyses performed by genotyping (real-time polymerase chain reaction) on smear slides with positive *Mycobacterium tuberculosis* culture. WT: wild type; MUT: mutated; POS: positive; NEG: negative; ND: not detected; UND: undetermined; UNR: unrealised; S: sensitive; R: resistant; qPCR: quantitative polymerase chain reaction; INH: Isoniazid.
